# Supplementary material for: Focal Accumulation of ROS Can Block Pyricularia oryzae Effector BAS4-Expression and Prevent Infection in Rice
Source: Int J Mol Sci. 2020 Aug 27;21(17):6196. doi: 10.3390/ijms21176196 (PMC7503722; doi:10.3390/ijms21176196)
Supplement: Supplementary file 1 [file ijms-21-06196-s001.zip › Figure S1.pdf]

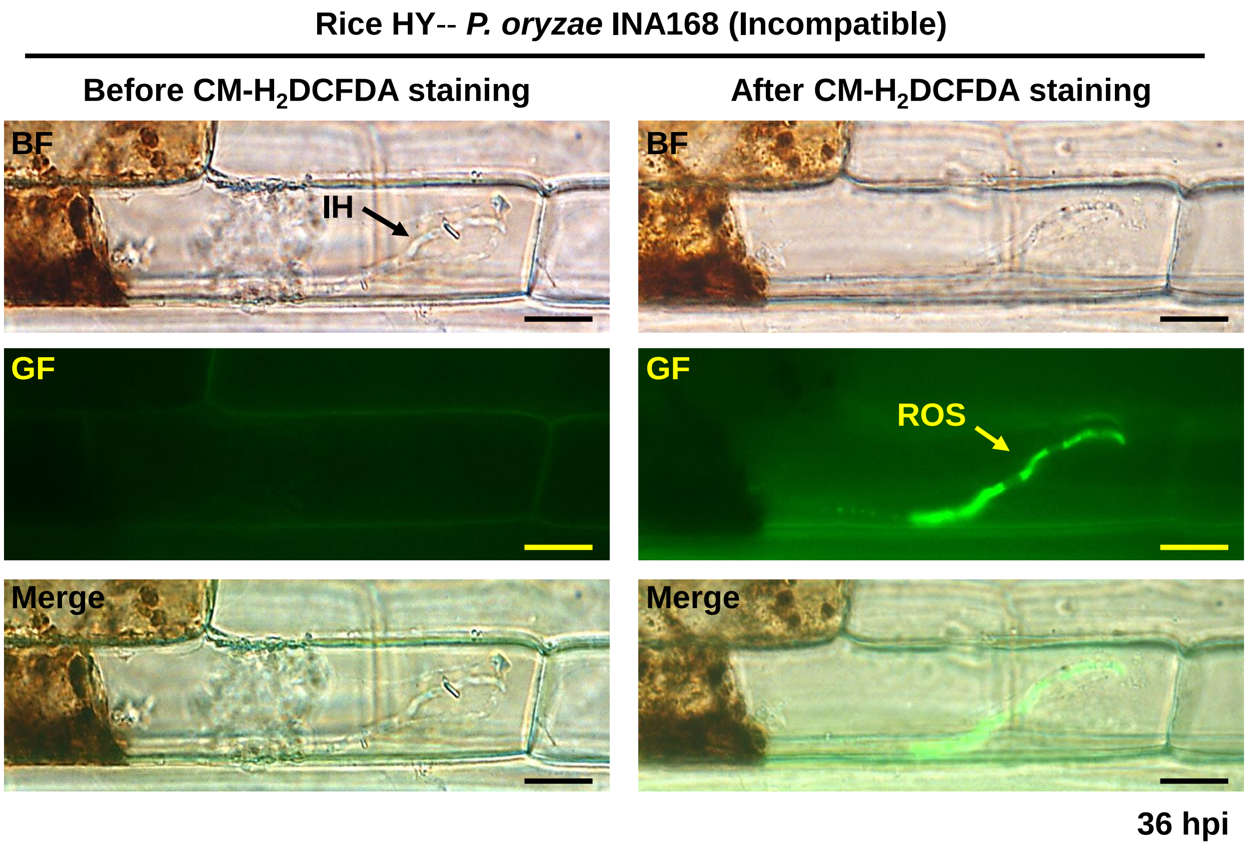
Rice HY-- P. oryzae INA168 (Incompatible)
Before CM-H2DCFDA staining
After CM-H2DCFDA staining
BF
IH
BF
GF
GF
ROS
Merge
Merge
36 hpi
