# Supplementary material for: Focal Accumulation of ROS Can Block Pyricularia oryzae Effector BAS4-Expression and Prevent Infection in Rice
Source: Int J Mol Sci. 2020 Aug 27;21(17):6196. doi: 10.3390/ijms21176196 (PMC7503722; doi:10.3390/ijms21176196)
Supplement: Supplementary file 1 [file ijms-21-06196-s001.zip › Table S1.pdf]

Table S1. Primers used in this study

Gene	Sequence		Description
OsRBOHB	Forward	GAATTCATGGCTGACCTGGAAGCAGGCA	Subcellular localization
	Reverse	CTCGAGTTAGAAGTTCTCCTTGTGGAAA	
OsRBOH RT	Forward	GGTTCCGACGTAACAAGCTC	qRT-PCR
	Reverse	ACTGCCCACTACGGTATCTG	
BAS4:eGFP	Forward	GTCGACTCGAGTTCGCTCGGGGGCTGG	INA168:BAS:eGFP mutant confirmation
	Reverse	GTCGACTCTAGTGATTCTCTAGATCAT	
Ubiquitin	Forward	GTGGTGGCCAGTAAGTCCTC	qRT-PCR
	Reverse	GGACACAATGATTAGGGATCA	
