# Supplementary figures and images for: Focal Accumulation of ROS Can Block Pyricularia oryzae Effector BAS4-Expression and Prevent Infection in Rice
Source: Int J Mol Sci. 2020 Aug 27;21(17):6196. doi: 10.3390/ijms21176196 (PMC7503722; doi:10.3390/ijms21176196)

**Rice HY--*P. oryzae* PO6-6  
(Compatible )**

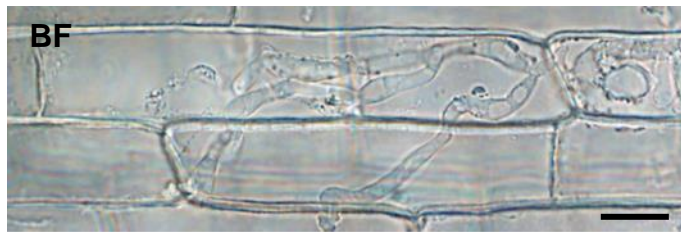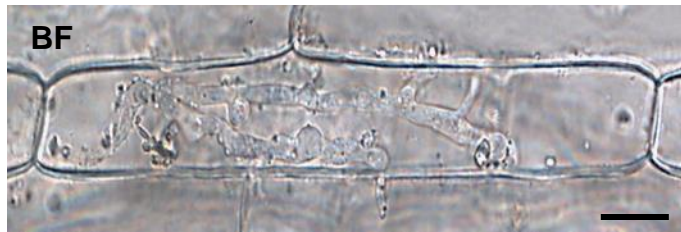

**Rice HY-- *P. oryzae* INA168  
(Incompatible )**

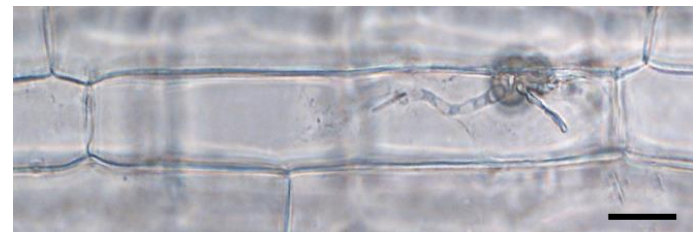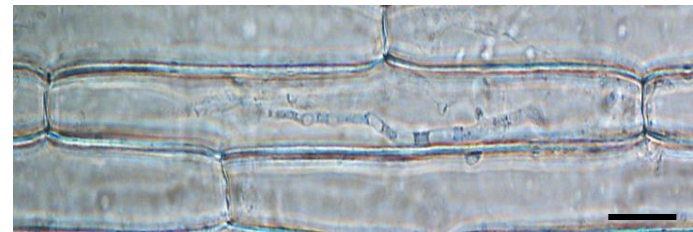

**36 hpi**

Supplement: Supplementary file 1 [file ijms-21-06196-s001.zip › Figure S2.pdf]

**A**Rice HY - *P. oryzae* INA168 (Incompatible)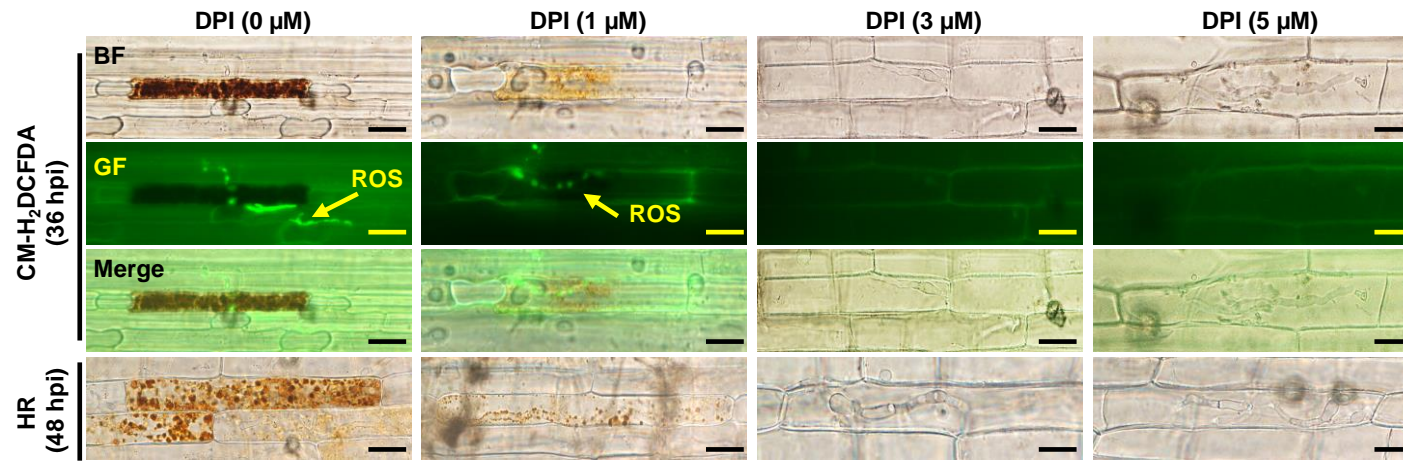**B**Rice HY - *P. oryzae* INA168:BAS4:eGFP (Incompatible)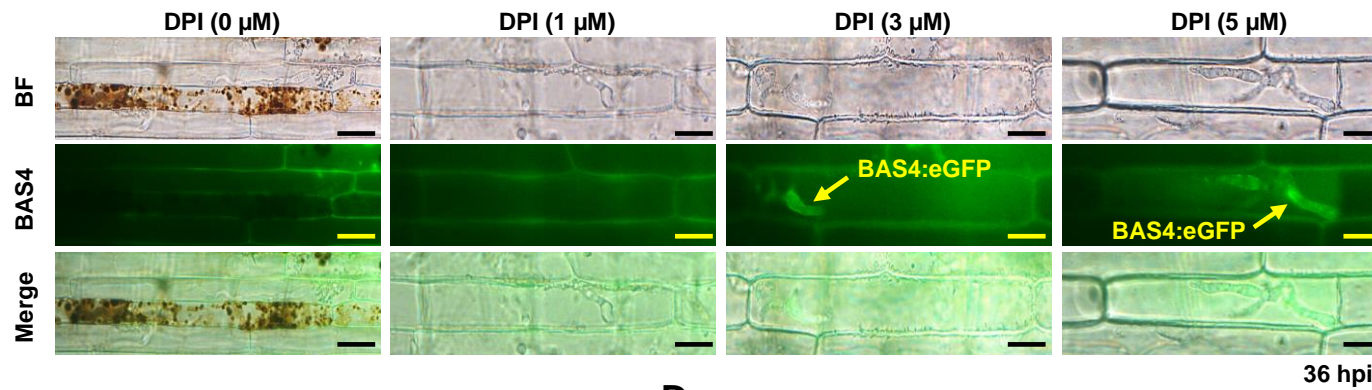**C**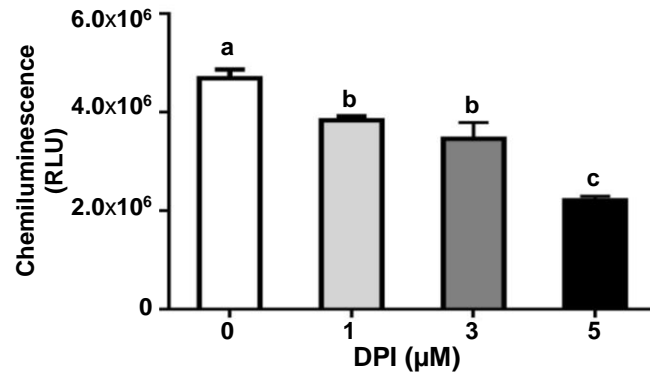**D**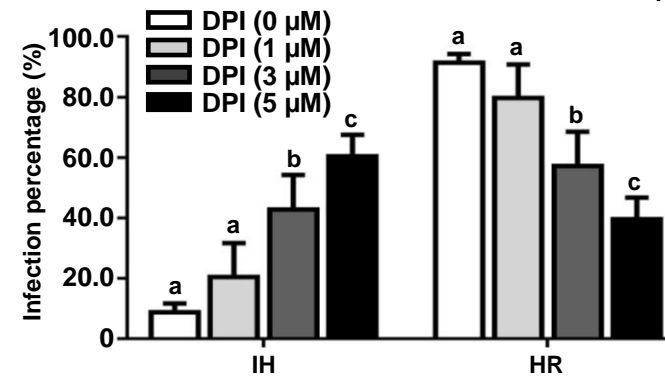

Supplement: Supplementary file 1 [file ijms-21-06196-s001.zip › Figure S3.pdf]
